# Structural analysis of the catalytic domain of Artemis endonuclease/SNM1C reveals distinct structural features

**DOI:** 10.1074/jbc.RA120.014136

**Published:** 2020-06-23

**Authors:** Md Fazlul Karim, Shanshan Liu, Adrian R. Laciak, Leah Volk, Mary Koszelak-Rosenblum, Michael R. Lieber, Mousheng Wu, Rory Curtis, Nian N. Huang, Grant Carr, Guangyu Zhu

**Affiliations:** 1 Discovery Biology, Albany Molecular Research Inc., Buffalo, New York, USA; 2 USC Norris Comprehensive Cancer Center, Departments of Pathology, Biochemistry & Molecular Biology, and Molecular Microbiology & Immunology, and the Molecular and Computational Biology Section of the Department of Biological Sciences, University of Southern California Keck School of Medicine, Los Angeles, California, USA; 3 Chemistry Department, Drug Discovery Division, Southern Research, Birmingham, Alabama, USA

**Keywords:** Artemis, SNM1C, endonuclease, crystal structure, SNM1 family, V(D)J recombination, DNA processing, hairpin opening, protein crystallization, protein expression, protein purification, protein structure

## Abstract

The endonuclease Artemis is responsible for opening DNA hairpins during V(D)J recombination and for processing a subset of pathological DNA double-strand breaks. Artemis is an attractive target for the development of therapeutics to manage various B cell and T cell tumors, because failure to open DNA hairpins and accumulation of chromosomal breaks may reduce the proliferation and viability of pre-T and pre-B cell derivatives. However, structure-based drug discovery of specific Artemis inhibitors has been hampered by a lack of crystal structures. Here, we report the structure of the catalytic domain of recombinant human Artemis. The catalytic domain displayed a polypeptide fold similar overall to those of other members in the DNA cross-link repair gene SNM1 family and in mRNA 3′-end-processing endonuclease CPSF-73, containing metallo-β-lactamase and β-CASP domains and a cluster of conserved histidine and aspartate residues capable of binding two metal atoms in the catalytic site. As in SNM1A, only one zinc ion was located in the Artemis active site. However, Artemis displayed several unique features. Unlike in other members of this enzyme class, a second zinc ion was present in the β-CASP domain that leads to structural reorientation of the putative DNA-binding surface and extends the substrate-binding pocket to a new pocket, pocket III. Moreover, the substrate-binding surface exhibited a dominant and extensive positive charge distribution compared with that in the structures of SNM1A and SNM1B, presumably because of the structurally distinct DNA substrate of Artemis. The structural features identified here may provide opportunities for designing selective Artemis inhibitors.

V(D)J recombination is the process by which T cells and B cells randomly assemble variable (V), diversity (D), and joining (J) gene segments to generate unique Igs and T cell receptors that can collectively recognize an almost infinite variety of different antigens (1). Artemis endonuclease is essential for the completion of this process (Fig. 1). It forms a complex with the DNA-dependent protein kinase catalytic subunit (DNA-PKcs), which phosphorylates Artemis to reveal a structure-specific endonuclease activity that opens the DNA hairpins generated by the RAG (recombination activation gene) complex during the V(D)J recombination process (2). Because the Artemis–DNA-PKcs complex is the only enzyme that is capable of opening DNA hairpins, loss-of-function mutants in either Artemis or DNA-PKcs block B and T cell maturation and increase the radiosensitivity of pre-B and pre-T cells. Humans and mice with loss-of-function Artemis gene mutations display severe combined immunodeficiency (SCID) (3). Artemis-deficient mice and DNA-PKcs-deficient mice exhibit a similar SCID phenotype and accumulate hairpin coding ends in thymocytes, suggesting that the Artemis–DNA-PKcs complex is the only enzyme that is capable of opening DNA hairpins (4).

**Figure 1.**
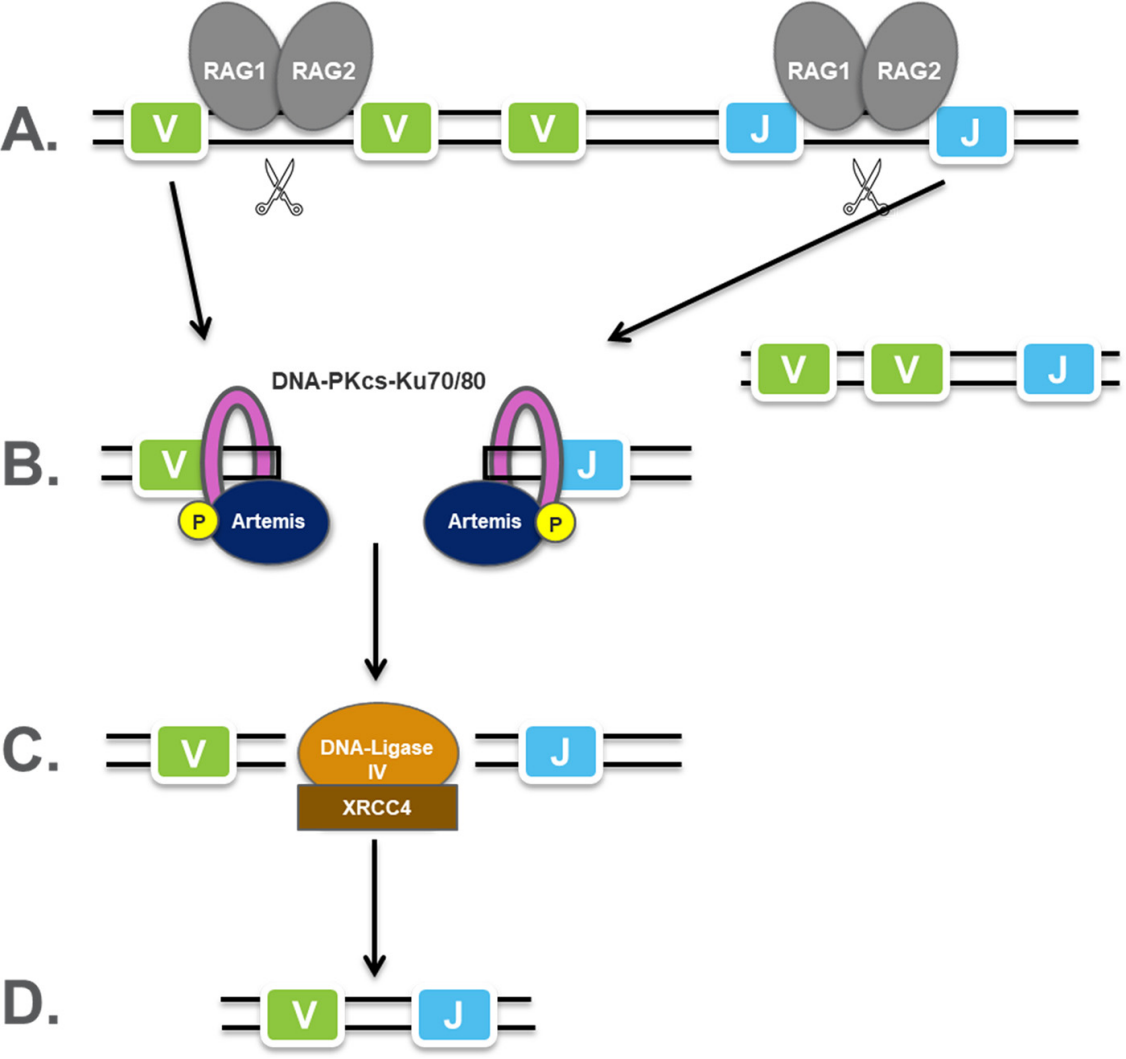
**The role of Artemis in V(D)J recombination.** *A*, using light-chain VJ recombination as an example, RAG1/RAG2 complexes (*gray ovals*) recognize recombination signal sequences between V and J segments and cleave the DNA to form hairpin ends. Unwanted V and J fragments are removed in this process. *B*, the Ku70/Ku80 complex recognizes the DNA end and recruits DNA-PKcs (*pink ring*) and Artemis (*navy oval*), which is phosphorylated by DNA-PKcs. Artemis opens the hairpin ends through its endonucleolytic activity and then processes DNA overhangs through its 5′-exonucleolytic or 5′-/3′-endonucleolytic activity. *C*, V and J segments are ligated together by ligase (*orange oval*). *D*, a VJ coding join is formed.

Human Artemis, 692 residues in length, contains an N-terminal catalytic domain (∼370 residues) with nuclease activity and a C-terminal regulatory region for DNA-PKcs interaction. The C-terminal segment (aa 448–462) is suggested to have a self-inhibitory role by interacting with the Artemis N-terminal catalytic domain (5). Artemis is activated through phosphorylation at multiple sites in the C-terminal region by DNA-PKcs, ataxia-telangiectasia mutated (ATM) kinase, and ataxia telangiectasia Rad3-related (ATR) serine/threonine kinases (6–8). In the context of V(D)J recombination, Artemis is activated through phosphorylation by DNA-PKcs, which is itself activated when bound to ligands with double-strand DNA (dsDNA) ends. The dsDNA ends include hairpinned DNA ends, which arise after RAG cutting of DNA. Based on amino acid sequence analysis, the N-terminal Artemis catalytic domain has been classified in the β-CASP (CPSF, Artemis, SNM1, and Pso) family with a β-CASP domain fused to a conserved metallo-β-lactamase fold (Fig. 2*B*). Within this family, there are a number of RNA nucleases, such as CPSF-73, *Bacillus* ribonucleases RNAS J1 and J2, and three human DNA nucleases, SNM1A, SNM1B/Apollo, and Artemis/SNM1C, which make up the SNM1 family (6, 7). The catalytic center is located in the metallo-β-lactamase domain and harbors metal ions for catalysis, for example, two zinc ions in the crystal structure of CPSF-73 (Fig. 2*C*) (8). Some common sequence motifs in this domain have been summarized, such as motifs I–IV (9) and motifs A–C. For Artemis, these motifs are the following: motif I, Asp-17; motif II, His-33, His-35, Asp-37, and His-38; motif III, His-115; motif IV, Asp-136; motif A, Asp-165; motif B, His-319; and motif C, V341 (Fig. 3) (7). The structure of human CPSF-73 reveals that motifs II, III, IV, and C directly coordinate to the metal center; motifs A and B form a hydrogen bond and may serve as a general acid for stabilizing the product during the catalytic reaction (8). Mutations of most motifs (except C) in Artemis have been analyzed. Except for His-38, these WT sequence residues have been proved to be critical and irreplaceable for the endonucleolytic activity of Artemis (10). The β-CASP domain is inserted in the metallo-β-lactamase domain. It creates the substrate binding pocket within the metallo-β-lactamase domain, and it is expected to confer substrate selectivity.

**Figure 2.**
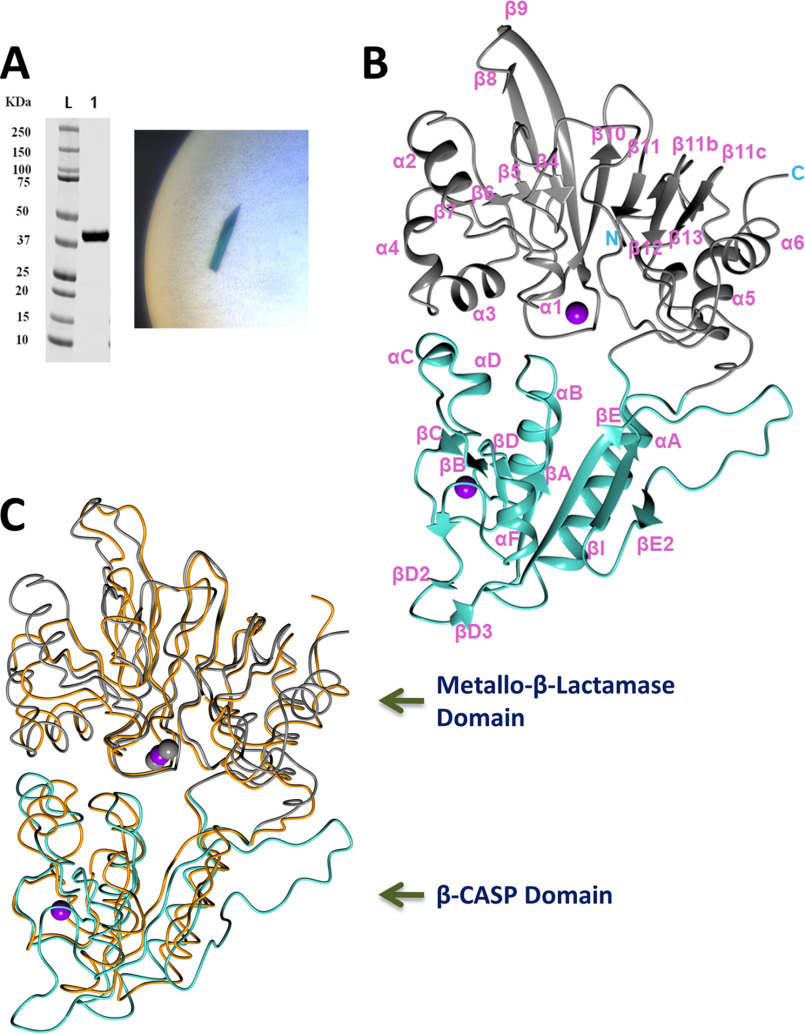
**Crystal structure of Artemis catalytic domain.** *A*, *left*, purified Artemis 1–368. *Right*, crystal of crystal form 1 with *blue color* from Cyanine5 dye. *B*, overall Artemis structure in ribbon diagram: the metallo-β-lactamase domain is colored *gray* and the β-CASP domain is colored *turquoise*. The zinc ions are shown in *purple spheres*. *C*, superposition of SNM1B/Apollo (PDB entry 5AHO) in *orange* for the polypeptide and gray spheres for zinc ions and Artemis colored as in *panel B*.

**Figure 3.**
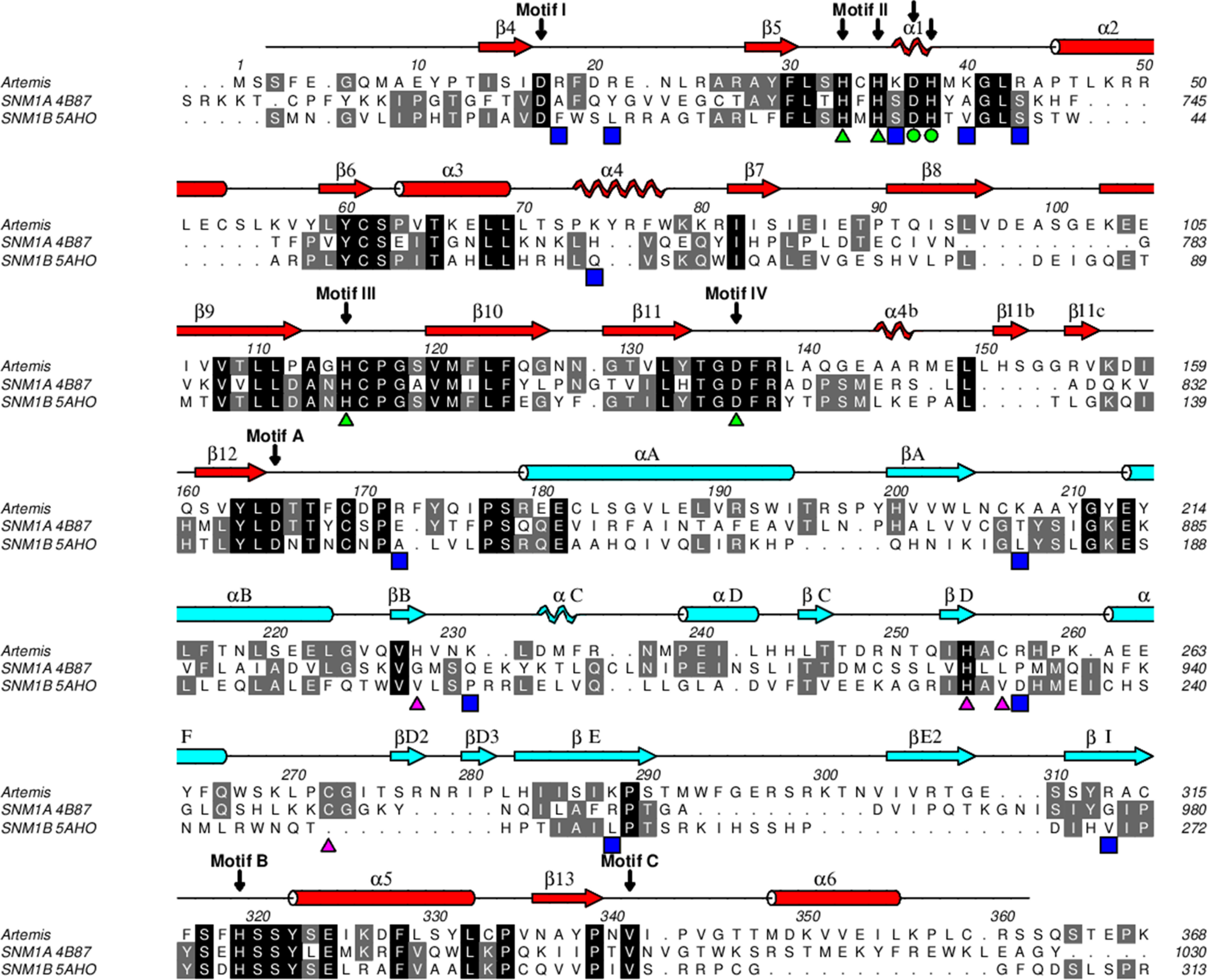
**Sequence alignment of human SNM1 family members.** The alignment of SNM1 family members was carried out with PROMALS3D (34), followed by manual adjustment, and displayed using ALINE (35). The secondary structure motifs were calculated by DSSP (36) and shown in *red* for the metallo-β-lactamase domain and *light blue* for the β-CASP domain. The α-helices are drawn as *cylinders*, β-strands as *arrows*, and 3_10_ helices as *helices*. The conserved active-site residues of SNM1 family are denoted with *green triangles* for zinc coordination and *green circles* for coordination of the second metal ion in the catalytic site. Residues that coordinate zinc in the β-CASP domain are marked with *pink triangles*. Other than His254, these residues are not conserved among SNM1 family members. *Blue squares* indicate positively charged residues in Artemis on the proposed substrate DNA binding surface.

To date, the crystal structures of CPSF-73 (8), SNM1A, and SNM1B (11) have been solved. Although Artemis shows less than 25% sequence identity to each of these proteins, it is anticipated to have a similar scaffold. As the only endonuclease in this family, Artemis is expected to exhibit different structural features, enabling it to process double-strand DNA substrates with various end structures (12, 13). Furthermore, the Artemis-DNA-PKcs complex is responsible for repairing dsDNA breaks, making this complex an attractive oncology drug target. Combining an inhibitor of Artemis or DNA-PKcs with targeted radiation therapy or treatment with a topoisomerase inhibitor should lead to increased sensitivity of the cancer cells to the DNA-damaging treatment. Cells deficient in Artemis are more sensitive than normal cells to X-rays (3). Inhibitors of DNA-PKcs have demonstrated efficacy in preclinical models and have been advanced into clinical trials (14, 15). Inhibitors of Artemis could be expected to be similarly effective, without some of the potential side effects that could arise from the inhibition of DNA-PKcs's other functions (16). As a member of the serine/threonine protein kinase family, DNA-PKcs is involved in multiple cellular processes, such as nonhomologous end joining, homologous recombination, cell cycle progression, and telomere maintenance (17), as well as V(D)J recombination. In contrast, Artemis has both a unique catalytic activity, DNA hairpin opening, and more limited roles in DNA recombination [V(D)J and nonhomologous end joining] than DNA-PKcs. To aid inhibitor design, an accurate Artemis structure is crucial. Here, we report the structure of the Artemis catalytic domain in two different crystal forms.

## Results

### The overall Artemis structure

The catalytic domain of Artemis (aa 1–368) was expressed and purified from insect cells. MS of the intact protein and peptide digestion with trypsin confirmed that the first methionine was removed and the second residue serine was fully acetylated. No other major posttranslational modification was observed. This is consistent with previous observations that Artemis has multiple phosphorylation sites at its C-terminal region and very few in its catalytic domain (18–20). Attempts to crystallize Artemis alone were unsuccessful; therefore, cocrystallization of Artemis with a number of DNA variants was attempted. Crystal hits of crystal form 1 were obtained when a 5′ overhang DNA was used, and the crystals were reproducible by microseeding. Crystal form 2 was obtained when a hairpin DNA was used. Crystals of both forms were dark blue, indicating that the different DNA ligands, each with a Cyanine5 dye tag, were present in the crystals (Fig. 2*A*). However, after the structures were solved, the DNA could not be built into the structures of either crystal form, which resulted in a higher *R*_work_/*R*_free_ gap during refinement. Some broken stacking electron density was observed around the 2-fold axis of crystal form 1 and a solvent channel in crystal form 2 (Fig. S1). The unsolved electron density in crystal form 2 was not in close proximity to the active site identified by the metal center. This indicated that the DNA was present in the solvent channel but bound promiscuously. In crystal form 1, a patch of unsolved electron density approaches the metal center in the active site. However, the majority of the DNA density does not interact with the surface of the protein, suggesting that it did not bind to the protein in a physiological way and likely bound promiscuously to promote crystallization. Furthermore, with the average of the 2-fold symmetry in crystal form 1, the DNA was not traceable in the current crystal form. Artemis could not be crystallized without the DNA even with microseeding, indicating that the DNA facilitated the crystal packing during crystallization even though it was not a uniform interaction.

Crystal form 1 contained one Artemis monomer in the asymmetric unit with 50% solvent (and disordered DNA). Residues 3–361 were built into the final refined model, along with two zinc ions. Crystal form 2 contained two Artemis molecules in the asymmetric unit and, for both molecules, residues 3–361, except residues 54, 299, and 300, were refined in the final structures. The three copies of Artemis were very similar to each other, with a root mean square deviation (r.m.s.d.) in the range of 0.37 to 0.60 Å (Fig. S2*A*). Because there was no major difference among these three copies, the highest-resolution Artemis structure in crystal form 1 is used here as the representative for illustration and discussion of structural features. Artemis exhibited the typical fold of the β-CASP family, with a metallo-β-lactamase domain and a β-CASP domain (Fig. 2*B*). Typical of the β-CASP family, the β-CASP domain encompassing residues 176–318 was inserted into the metallo-β-lactamase domain. About 280 Cα atoms of Artemis can be superposed to DNA nucleases SNM1A and SNM1B with an r.m.s.d. of 2.2 Å for both superpositions (Fig. 2*C*) (11). Directly superposing Artemis and RNA nuclease CPSF-73 (2I7T) gave an r.m.s.d. of 3.4 Å over 244 Cα atoms; when the metallo-β-lactamase and the β-CASP domains were superposed separately, the r.m.s.d. was reduced to 2.2 Å for both domains, over a total of 261 Cα atoms from the two domains. This indicates that the relative orientation between the two domains is conserved within the SNM1 family but is different from those of other RNA nucleases.

Artemis shares less than 25% sequence identity with SNM1A or SNM1B. From a structure-based alignment (Fig. 3), the three proteins in the SNM1 family share a higher similarity within the metallo-β-lactamase domain. The registration of secondary structure motifs followed the nomenclature of CPSF-73. The structures of Artemis and CPSF-73 contain α2 (aa 45–53) and α4 (aa 73–78) helices, whereas these two regions are shorter and do not form helices in SNM1A and SNM1B. Similar to SNM1A, but not SNM1B, Artemis has an α6 (aa 348–358) helix at the C terminus, although the loop connecting β13 (aa 336–339) and α6 points in different directions (11). At the N-terminal region, SNM1B has a β3 strand that forms an anti-parallel β-sheet with β4. The N termini of Artemis (aa 5–8) and SNM1A are located at the same position but did not adopt a β-strand conformation; instead, SNM1A forms an α helix. In the β-CASP domain, Artemis residues 291–309 are positioned differently, forming a long loop and βE2 strand (aa 304–308), which is antiparallel to βI (aa 311–315). This β strand was not present in SNM1B or CPSF-73, in which this region folded toward the center of the protein. SNM1A also might extend this corresponding region outwards, as seen in the Artemis structure (Artemis residues 291–309), but 10 of the relevant residues in the SNM1A structure were disordered. The region of Artemis from 258–283 is also structured differently, coordinating an unanticipated structural zinc, which is described in more detail in the next section. All aforementioned differences from SNM1A and SNM1B were located on one side of Artemis, suggesting that this is the putative substrate binding surface.

### Zinc binding sites

**Figure 4.**
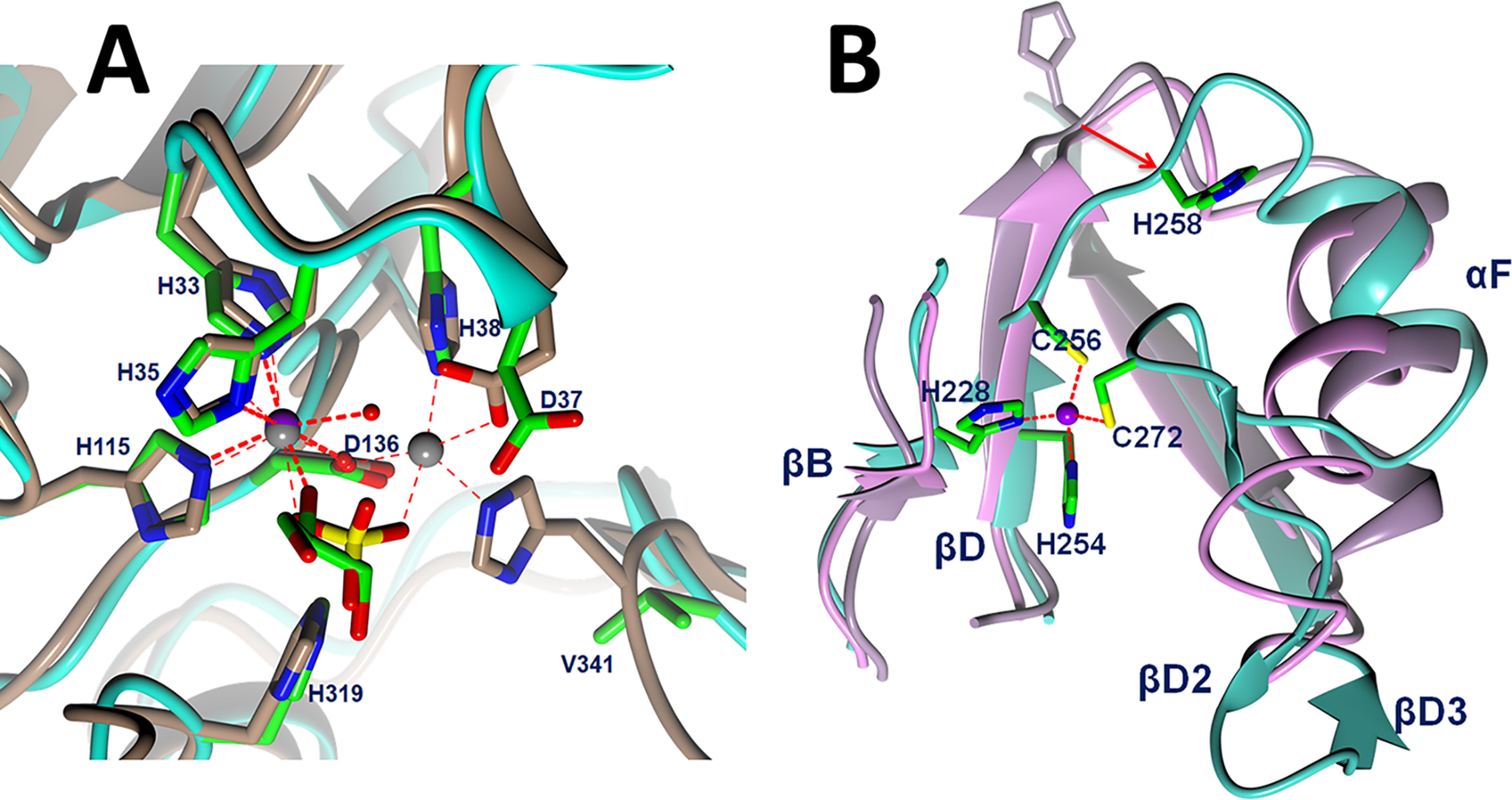
*A*, comparison of active site of Artemis with CPSF-73 (PDB entry 2I7T) (8). Zinc ions are shown as a *purple sphere* for Artemis and *gray spheres* for CPSF-73. Artemis backbone is shown as a *cyan ribbon*, and the side chains of conserved residues are shown in *green* for carbon, *blue* for nitrogen, and *red* for oxygen atoms. Water molecules are shown as *red spheres*. The main chain and side chains of CPSF-73 are shown in *brown*. A sulfate (*yellow sulfur*) ion in the CPSF-73 structure and glycerol in Artemis are bound above the catalytic metals in a position likely occupied by the scissile phosphodiester of the substrate. *B*, novel, structural zinc binding site in Artemis β-CASP domain induced structural differences among Artemis in *cyan*, SNM1A in *pink*, and SNM1B/Apollo in *lilac*. An 8.5-Å movement from SNM1B/Appolo His-234 to Artemis His-258 is elicited as a *red arrow*.

Two zinc ions were located unambiguously with anomalous diffraction data (Fig. S3). One zinc ion is located at the active site with octahedral coordination by residues His-33, His-35, His-115, Asp-136, a water molecule, and a glycerol molecule (Fig. 4*A*). His-115 and Asp-136 belong to conserved motif III and IV, respectively; His-33 and His-35 belong to motif II, with the characterized sequence HxHxDH for the metallo-β-lactamase family (3). These four residues are conserved in the SNM1 family, and mutating any one of them abolishes *in vitro* and *in vivo* functions of Artemis (10). Artemis did not bind a second zinc ion in the active site, as seen with CPSF-73 (8). Although the residues Asp-37 and His-38 in motif II are still conserved and located at the same positions as CPSF-73, the zinc coordinating ligand His-418 in motif C of CPSF-73 was replaced by Val-341 in Artemis, and the Cα was shifted 1.7 Å away from the active site. Val-341 is conserved in the SNM1 family; thus, losing a metal-binding ligand is a common feature for the family. The Cα positions of this valine in SNM1A and SNM1B are similar to that of CPSF-73. There was only one zinc ion located in the active site of SNM1A, similar to Artemis. Mutating the valine to histidine in SNM1A did not change activity and substrate selectivity (21). Two zinc ions were observed in the SNM1B structure; however, it was stabilized by tartrate in the crystallization buffer. Motif B His-319 in Artemis aligned well with His-396 in CPSF-73 (Fig. 4*A*), which was proposed to be a general acid to stabilize the leaving group in the hydrolysis reaction (8). Similar to CPSF-37, His-319 forms a hydrogen bond with the side chain of Asp-165 (motif A). Both residues were confirmed to be critical to Artemis's endonuclease function (10).

The second zinc ion is located in the β-CASP domain, where it appears to have a structural role. This feature is unique to Artemis, as other β-CASP proteins do not have a zinc binding site within this domain. This zinc has tetrahedral coordination by residues His-228, His-254, Cys-256, and Cys-272 (Fig. 4*B*). Residues His-228, His-254, and Cys-256 are found on two parallel β-strands, βB and βD, which are located at similar positions in other members in the SNM1 family. Because of the coordination of Cys-256, the loop between βD and αF is shifted. As an example, Cα of His-258 moved 8 Å outward from its equivalent position in SNM1A and SNM1B. The fourth coordination partner, Cys-272, is located on an inserted β-turn, which is not present in SNM1A, SNM1B, or CPSF-73. The restructuring of this loop and the coordination of Cys-256 induced a ∼40° rotation of helix αF from its position in SNM1A and SNM1B (Fig. 4*B*). Most residues of this zinc binding motif were not conserved within the SNM1 family. However, they are conserved in Artemis across species, suggesting an important but unidentified function for this structural feature. His-254 is conserved within the SNM1 family and was previously proposed to be involved in metal ion coordination at the active site. Mutation of His-254 disrupted the *in vivo* and *in vitro* function of Artemis (22), indicating that this unique zinc binding motif in the β-CASP domain of Artemis is both structurally and functionally important.

### Putative DNA substrate binding site

**Figure 5.**
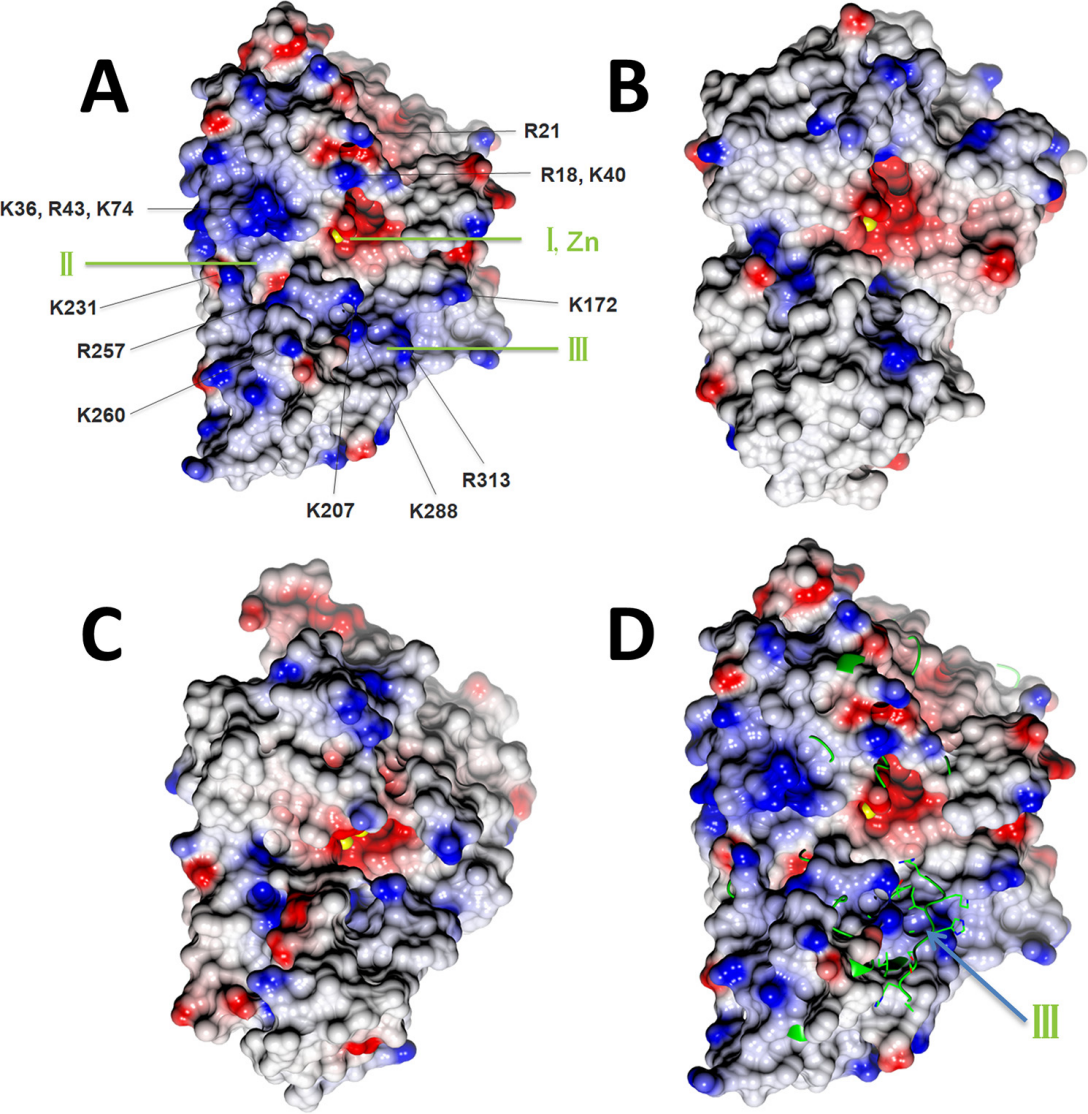
**Surface representation with electrostatic potential distribution of SNM1 family members in the same orientation.** Metal ions at the active site are drawn as *yellow spheres*. *A*, Artemis/SNM1C oriented with pockets I, II, and III visible; note the abundance of (*blue*) positively charged residues in the vicinity of the catalytic site that may contribute to DNA substrate binding. *B*, SNM1A. *C*, SNM1B/Apollo. *D*, Artemis is shown as a surface model, and SNM1B/Apollo is shown as a *yellow ribbon* and *cylinder* (for side chain of region 259–268). In SNM1B/Apollo, pocket III is not open and is instead occupied by loop 259–268. The color intensity corresponds to the calculated electrostatic potential, from −20 kT/e (*red*) to +20 kT/e (*blue*).

Because the metal ions form the catalytic center for nucleases, the same surface was proposed to be the active site for DNA binding and cleavage. With zinc binding in the β-CASP domain and multiple reorientations within both domains, the active site of Artemis exhibited a molecular surface different from those of SNM1A and SNM1B (Fig. 5). The catalytic zinc ion bound to pocket I (Fig. 5*A*), which is located between the metallo-β-lactamase and β-CASP domains. Side pocket II is partially separated from pocket I by the main chain of Ala-208 and Ala-209. Both pocket I and II also exist in SNM1A and SNM1B. It has been proposed that this region fits the groove of the DNA substrate for other SNM1 family members (11). In Artemis, there was an additional pocket III below pocket I, which formed in the β-CASP domain by the reorientation of residues 292–309 (forming βE2) and residues 256–281 produced by coordination of residues to the structural, noncatalytic zinc ion within the β-CASP domain. The active-site surface of Artemis exhibited a dominant positive charge distribution, including residues Arg-18, Arg-21, Lys-36, Lys-40, Arg-43, Lys-74, Arg-172 Lys-207, Lys-231, Arg-257, Lys-260, Lys-288, and Arg-313. These residues are conserved in Artemis across species and might recognize negatively charged DNA as the substrate. These positively charged residues are also unique to Artemis within the SNM1 family (Fig. 3). As a result, SNM1A and SNM1B do not show as extensive positively charged patches as Artemis (Fig. 5).

## Discussion

We have solved the structure of Artemis endonuclease from cocrystals with two different DNA substrates, a 5′ overhang and a DNA hairpin. We found that Artemis displays an overall fold similar to that of other members of the SNM1 family and CPSF-73 but also shows unique features, such as a novel zinc binding site in the β-CASP domain and a distinct substrate binding surface. DNA nucleases in the SNM1 family, including SNM1A, SNM1B/Apollo, and Artemis/SNM1C, exhibit a wide open substrate binding pocket (pockets I and II) compared with RNA nuclease CPSF-73. It is likely that this allows bulky DNA substrates to fit into this pocket. Artemis also extends its substrate binding site to pocket III, which appears to be deeper and wider than those of SNM1A and SNM1B. In addition, more positively charged residues are clustered on this surface. Artemis is the only known endonuclease in the SNM1 family, because SNM1A and SNM1B are confirmed to exhibit only exonuclease activity. Artemis is able to process several different DNA substrate structures, such as blunt ends, hairpin ends, 5′ overhangs, 3′ overhangs, and symmetrical bubble DNA structures (13). This requires that Artemis has an additional substrate recognition pocket to restrict and fold the DNA ends. Our structure indicates that pocket III serves such a function.

The novel zinc binding site in the β-CASP domain lies in close proximity to pockets II and III. Moreover, coordination of the zinc ion by His-228, His-254, Cys-256, and Cys-272 contributes to structural changes in Artemis, leading to the creation of both pockets II and III. For instance, zinc coordination by Cys-256 and Cys-272 results in the rearrangement of residues between these two cysteine residues compared with SNM1A and SNM1B, including helix αF (Fig. 4*B*). The side chain of Arg-257 points directly into pocket II, and another positively charged residue, Lys-260, is located at the edge of pocket III (Fig. 5*A*). Furthermore, mutation of His-254 eliminates Artemis activity *in vitro*, and inherited mutations of His-254 result in radiosensitive severe combined immune deficiency in humans (22), suggesting that the 3D structure imposed by zinc binding at the novel zinc binding site is critical for the functional activity and substrate selectivity of Artemis.

While we were preparing this manuscript, an Artemis structure (PDB entry 6TT5) was deposited and released by Gileadi's group. Whereas the crystal form of 6TT5 is different from both of those that we report here, the overall structures of the Artemis protein were very similar. Superposition of the 3 structures aligned 347 Cα (out of 348 residues in 6TT5) with an r.m.s.d. of 0.68 Å (Fig. S2*B*). Therefore, this PDB confirmed the structural changes we observed for Artemis compared with those of other SNM1 family members. Furthermore, it showed the relevant positions between the metallo-β-lactamase and the β-CASP domains were unchanged. Although the resolution of this structure was higher (1.5 Å *versus* 2.0 Å), 6TT5 was missing residues 259–268, which included helix αF in our structure. This helix connected two zinc binding motifs in the β-CASP domain and packed to the structure mainly through hydrophobic interaction with a packing surface of 366 Å^2^. The same position of helix αF was also observed for the structures from crystal form 2. The position of αF in our structures could not be fitted into 6TT5 because of the steric hindrance from the crystal packing. Therefore, the disordered αF in 6TT5 likely resulted from a crystallization artifact.

One zinc and one nickel ion were located at the active site in the metallo-β-lactamase domain in 6TT5. A nickel ion replaced the zinc ion in the active site of our structure and coordinated six ligands with octahedral geometry. Four ligands were residues His-33, His-35, His-115, and Asp-136, and the other two ligands were a water molecule and an ethylene glycol molecule. A second metal ion present on 6TT5, which was not present in our structure, was a zinc ion coordinated to five ligands with a square pyramidal geometry. Three ligands were protein residues Asp-37, His-38, and Asp-136, and the other two ligands were water molecules. The dimetal center in 6TT5 was similar to that of CPSF-73, in which there are six coordinating ligands from the protein. His-418 in CPSF-73 in motif C is occupied by Val-341 in Artemis. This replacement contributed to the low-affinity binding for the second metal ion in our structure. However, we also observed that a divalent metal, such as magnesium or nickel, could be soaked into this position under certain conditions, with Asp-37 rotating to coordinate with the metal ion (unpublished results). This indicates that although Artemis was missing one important metal binding motif, the dimetal center may still form during catalytic reaction, presumably stabilized by the DNA substrate. According to the catalytic mechanism of nucleases, three major classes exist based on metal ion dependence: requiring none, one, or two metal ions for catalysis (23). Interestingly, mutating Asp-37 in motif II of Artemis abolished the endonuclease function; however, mutant H38A in the same motif maintained 50% activity of WT Artemis in an *in vivo* assay (10). Along with the crystal structure, this indicated that the binding of a second metal ion is important during catalysis, but a stable binding was no longer required. If a dimetal center is formed for catalysis, magnesium may be used instead of zinc. Artemis was active in a magnesium-containing buffer but was inactive in buffers containing zinc or nickel (2, 24). Intracellular concentrations of free magnesium (0.5–1 mM) (25, 26) are consistent with the role for magnesium as the physiologically relevant second metal ion employed during catalysis at the catalytic site of Artemis.

As Artemis is a potential oncology drug target, these structures provide valuable information for *in silico* inhibitor design. As the metal ions in the active site are critical for nuclease activity, compounds designed to coordinate with a divalent metal ion and bind at pocket I may provide an effective approach for the development of a potent Artemis inhibitor. Our structures show the surface and structural features of pockets II and III of Artemis are unique in the SNM1 family, presenting a distinct molecular surface and charge distributions for small-molecule interaction. Compared with the relatively wide open pocket I, pockets II and III may provide enhanced opportunities for inhibitor design and selectivity.

## Experimental procedures

### Artemis expression and purification

Human Artemis (aa 1–368) was prepared from a baculovirus construct in Sf9 cells. The plasmid containing codon-optimized Artemis (aa 1–368) with a C-terminal tobacco etch virus (TEV) cleavage site and a 6× His tag was cloned into a pFastBac/CT-TOPO vector. A C-terminal tag, KGENLYFQGHHHHHH, was appended to the native sequence to provide a TEV protease-cleavable His tag. Sf9 cells were infected for 48 h at 27 °C. The cells were harvested by centrifugation, resuspended at 4 °C in lysis buffer [20 mm NaH_2_PO_4_, 500 mm NaCl, 10% glycerol, 20 mm imidazole, and 0.5 mm tris(2-carboxyethyl)phosphine (TCEP), pH 7.6] containing EDTA-free protease inhibitor mixture (Roche) and 0.5 mm PMSF, and lysed by a Microfluidizer (Microfluidics) at 10,000 psi for 2 passes. The lysate was centrifuged at 17,500 × *g* at 4 °C for an hour, and the supernatant was mixed with Ni-Sepharose 6 Fast Flow resins (GE Healthcare) at a ratio of 1.5 ml resin per liter of cells. After gentle shaking at 4 °C for an hour, the resins were washed in an Econo column (Bio-Rad) with 20 column volumes of wash buffer (20 mm NaH_2_PO_4_, 500 mm NaCl, 10% glycerol, 50 mm imidazole, and 0.5 mm TCEP, pH 7.6), and the protein was eluted with elution buffer (20 mm NaH_2_PO_4,_ 500 mm NaCl, 10% glycerol, 250 mm imidazole, and 0.5 mm TCEP, pH 7.6). Fractions containing Artemis were pooled and treated with TEV protease at a ratio of 1:20 (w/w) (Molecular Cloning Laboratories) and dialyzed overnight against dialysis buffer (20 mm NaH_2_PO_4_, 500 mm NaCl, 10% glycerol, and 5 mm DTT, pH 7.6) at 4 °C. The untagged protein was concentrated using a 10-kDa-cutoff centrifugal concentrator and applied to a HiLoad 16/600 Superdex 200-pg (GE Healthcare) size exclusion column in dialysis buffer. The fractions containing Artemis were pooled, and the salt concentration was adjusted to ∼200 mm with 20 mm HEPES, pH 7.6. The Artemis sample was loaded onto a Resource S column (GE Healthcare) and eluted with a 20 column volume gradient of NaCl (200–750 mm in 20 mm HEPES, pH 7.6). Artemis eluted at ∼450 mm NaCl and was then pooled, concentrated to ∼1 mg/ml, flash-frozen, and stored at −80 °C. Protein concentration was measured with absorbance at 280 nm using an extinction coefficient of 1.26 (mg/ml)^−1^ cm^−1^; a typical yield was 1–1.5 mg per liter of Sf9 culture. Protein with a His tag was purified with the same procedure, excluding the TEV cleavage step. The molecular weight and posttranslational modification of Artemis with His tag was confirmed by MS (MSBioworks). To obtain the molecular mass of the intact protein, 1 μg of His-Artemis was analyzed by LC-MS using an X-Bridge BEH C_4_ column, 2.1 mm by 50 mm (Waters), interfaced to a Q Exactive mass spectrometer (Thermo Scientific). Data were acquired from 600–2000 *m*/*z* at a resolution of 17,500 full width at half maximum (FWHM) (at 400 *m*/*z*) with three microscans per spectrum. To identify posttranslational modifications, 6× His-tagged Artemis was digested with trypsin, and the digestion product was analyzed by nano LC-MS/MS with a Waters NanoAcquity HPLC system interfaced to a Q Exactive mass spectrometer. Peptides were loaded on a trapping column and eluted over a 75-μm analytical column at 350 nl/min; both columns were packed with Luna C_18_ resin (Phenomenex). The mass spectrometer was operated in data-dependent mode, with the Orbitrap operating at 70,000 FWHM and 17,500 FWHM for MS and MS/MS, respectively. The fifteen most abundant ions were selected for MS/MS.

### Crystallization of Artemis

Artemis protein with the His tag removed was crystallized using the hanging-drop vapor diffusion method at 23 °C. To generate crystals of crystal form 1, the protein sample at 1 mg/ml was mixed with annealed overhang DNA (5′-[Phos]CACAGCTGATCGC-3′ and 5′-[Cyanine5]GCGATCAGCT-3′) at a molar ratio of 1:0.6 (protein:DNA). The protein-DNA mixture was then concentrated to ∼5 mg/ml Artemis using a 10-kDa-cutoff centrifugal concentrator. Hanging drops were prepared by mixing 1 μl Artemis-DNA and 1 μl reservoir solution containing 50 mm MES, pH 6.5, 0.1 M LiCl, 0.01 M MgCl_2_, 12% (w/v) PEG 4000. The drops were seeded with Artemis microcrystals 24 h after vapor diffusion. The crystals grew to ∼0.1 by 0.4 mm over a 2-week period before harvesting for analysis. Crystal form 2 was obtained in a similar way using hairpin DNA (5′-[Cyanine5]CGCGGT*G*T*CCGCG-3′ [an asterisks indicates phosphorothioate bond modification]) with a protein:DNA ratio of 1:1.2. The reservoir solution contained 50 mm MES, pH 6.5, 0.1 m NaCl, 0.05 m LiCl, 0.01 m MgCl_2_, 17% (w/v) PEG 4000. For both crystal forms, the crystals were transferred to the reservoir solution supplemented with 15% (v/v) glycerol for cryoprotection and then flash-frozen in liquid nitrogen for data collection.

### Data collection and structure determination

Diffraction data were collected at the IMCA-CAT beamline 17ID (crystal form 1) or GMCA-CAT beamline 23IDD (crystal form 2) at the Advanced Photon Source at Argonne National Laboratory using a Pilatus 6M detector. Single-wavelength anomalous diffraction (SAD) data of crystal form 1 were collected at the zinc K-edge, and the native data were collected to 1.97-Å resolution. The diffraction data of crystal form 2 were collected to 2.43 Å. The data were processed with Xia2 (27), XDS (28), DIALS (29), and Aimless (30). The phases were solved using the SAD data by Crank, and manual model building was carried out using Coot. The structures for both crystal forms were solved by molecular replacement using the structure from SAD data as the searching model by Phaser (31), and the structures were subsequently refined using Refmac5 (32). The crystallographic figures were generated by CCP4MG (33). The crystallographic statistics are summarized in Table 1.

**Table 1:** **Crystallography data collection and refinement statistics**

Parameter	Value(s) for:		
	SAD	Crystal form 1	Crystal form 2
**PDB ID**		6WO0	6WNL
**Data collection**			
Beamline	APS 17-IDD	APS 17-IDD	APS 23-IDD
Wavelength (Å)	1.28217	1.00	1.033
Space group	P2_1_2_1_2	P2_1_2_1_2	P2_1_2_1_2_1_
**Unit cell dimensions**			
*a* (Å)	72.74	72.81	69.65
*b* (Å)	111.01	111.00	105.68
*c* (Å)	54.85	55.17	111.06
α = β = γ (°)	90	90	90
Resolution (Å)	65–2.37	40–1.97	50–2.37
Highest-resolution shell (Å)	2.43–2.37	2.02–1.97	2.43–2.37
No. of total reflections	77,277	204,774	226,744
No. of unique reflections	18,714	32,168	35,315
*R*_merge_ (%)	6.7 (36.5)*^a^*	5.0 (82.8)	14.8 (78.4)
*I*/σ(*I*)	9.2 (2.3)	16.0 (2.4)	9.7 (2.4)
Completeness (%)	93.8 (94.4)*^b^*	99.6 (99.6)	100.0 (99.6)
Multiplicity	2.1 (2.2)*^b^*	6.4 (6.6)	6.4 (6.5)
**Refinement**			
*R*_work_		0.218 (0.336)	0.221 (0.263)
*R*_free_		0.270 (0.370)	0.280 (0.309)
No. of protein atoms		2921	5784
No. of zinc ions		2	4
No. of solvents		117	207
Avg B-factor (Å^2^)			
Protein		57.6	34.0
Zinc (Å^2^)		44.4	27.1
Solvent (Å^2^)		51.7	32.1
r.m.s.d. bond length (Å)		0.013	0.005
r.m.s.d. angle (°)		1.95	1.33
**Ramachandran plot (%)**			
Allowed		97.5	97.9
Generous		2.5	2.1
Disallowed		0.0	0.0

*^a^* The values in parentheses are for the highest-resolution shell.

*^b^* For anomalous completeness and multiplicity.

## Data availability

Coordinates and the structural factors of Artemis have been deposited in the PDB under codes 6WO0 (crystal form 1) and 6WNL (crystal form 2). All other data are contained within the manuscript.

## Supplementary Material

Supporting Information
