# Supplementary material for: Structural analysis of the catalytic domain of Artemis endonuclease/SNM1C reveals distinct structural features
Source: J Biol Chem. 2020 Jun 23;295(35):12368–77. doi: 10.1074/jbc.RA120.014136 (PMC7458816; doi:10.1074/jbc.RA120.014136)
Supplement: Supporting Information [file supp_295_35_12368__index.html]

Structural analysis of the catalytic domain of Artemis endonuclease/SNM1C reveals distinct structural features — Crystal structure of Artemis — Structural analysis of the catalytic domain of Artemis endonuclease/SNM1C reveals distinct structural features — Crystal structure of Artemis — Supporting Information 

# Structural analysis of the catalytic domain of Artemis endonuclease/SNM1C reveals distinct structural features

## Supporting Information

- Supporting Information (to be published online) - Supporting figures
