# Supplementary material for: Structural analysis of the catalytic domain of Artemis endonuclease/SNM1C reveals distinct structural features
Source: J Biol Chem. 2020 Jun 23;295(35):12368–77. doi: 10.1074/jbc.RA120.014136 (PMC7458816; doi:10.1074/jbc.RA120.014136)
Supplement: Supporting Information [file supp_RA120.014136_160515_3_supp_551826_qc76zp.pdf]

Supplemental Information

(A)

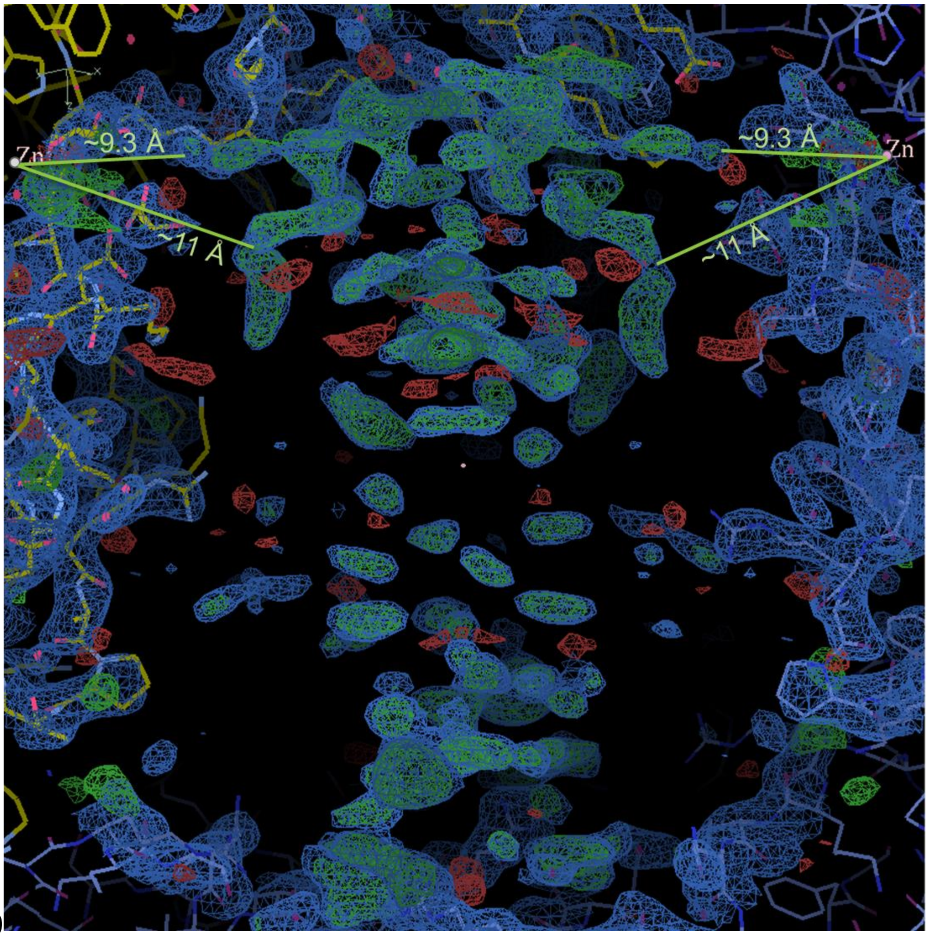

(B)

(C)

(D) 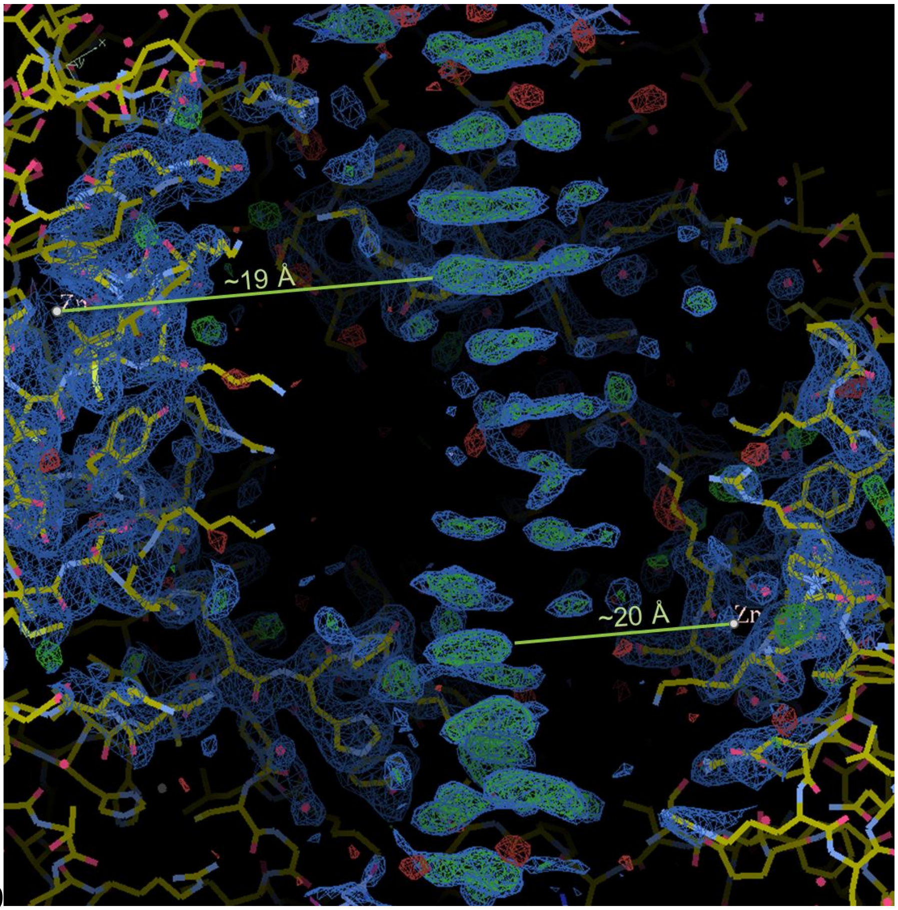

Figure S1 Unmodeled electron density of overhang DNA in the solvent channel for crystal form 1 **(A, B)** and hairpin DNA in crystal form 2 **(C, D)**. The *2Fo-Fc* map is shown in blue and contoured at 1.0σ, and the *Fo-Fc* maps are shown in green and red and contoured at +/- 3.0σ. Active site zinc ions are labeled and proximity to unmodeled DNA density are shown (B and D).

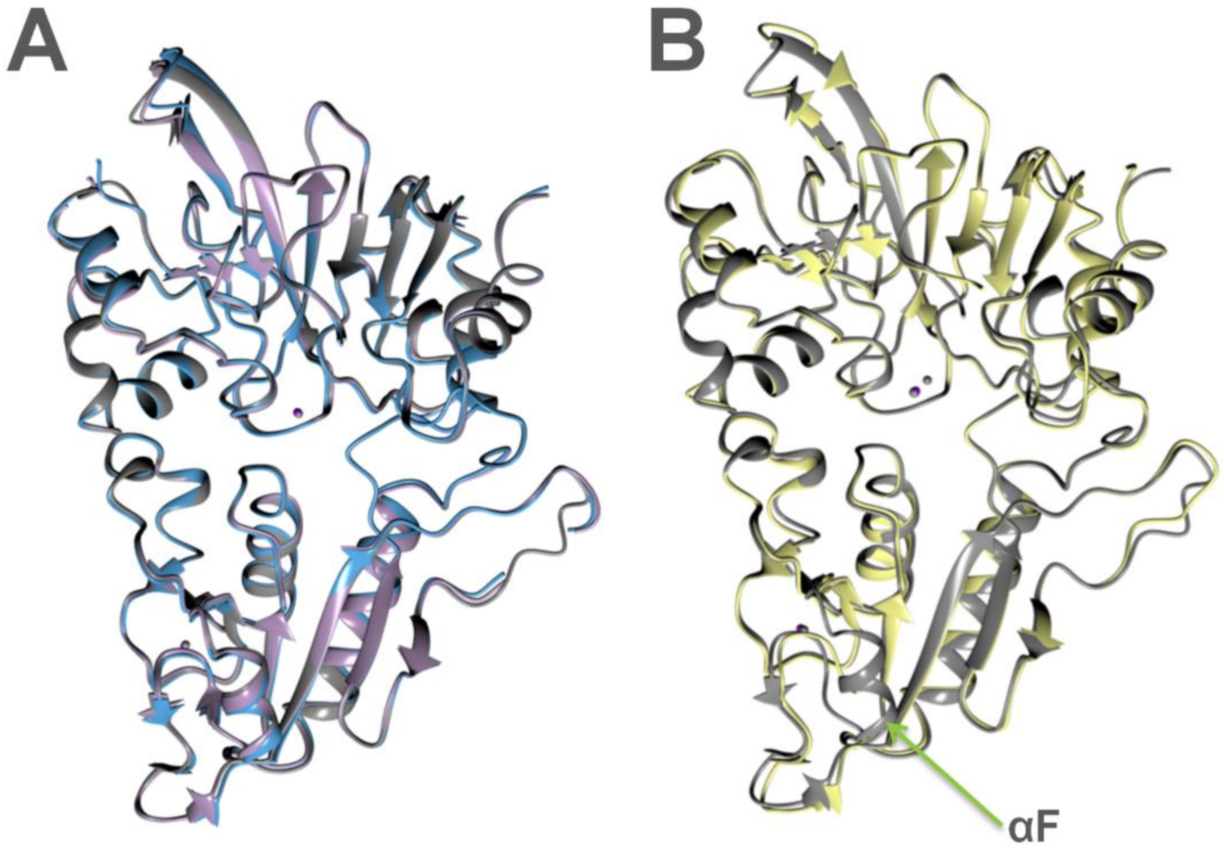

Figure S2 **(A)** Superposition of Artemis structures from crystal form 1 (grey), molecule A of crystal form 2 (lilac), and molecule B of crystal form 2 (light blue). The root mean square deviations of Cα are 0.37Å (form 1 – A of form 2), 0.60Å (form 1 – B of form 2), and 0.50 (A – B of form 2). **(B)** Superposition of Artemis structure from crystal form 1 (grey) and PDB 6TT5 (yellow).

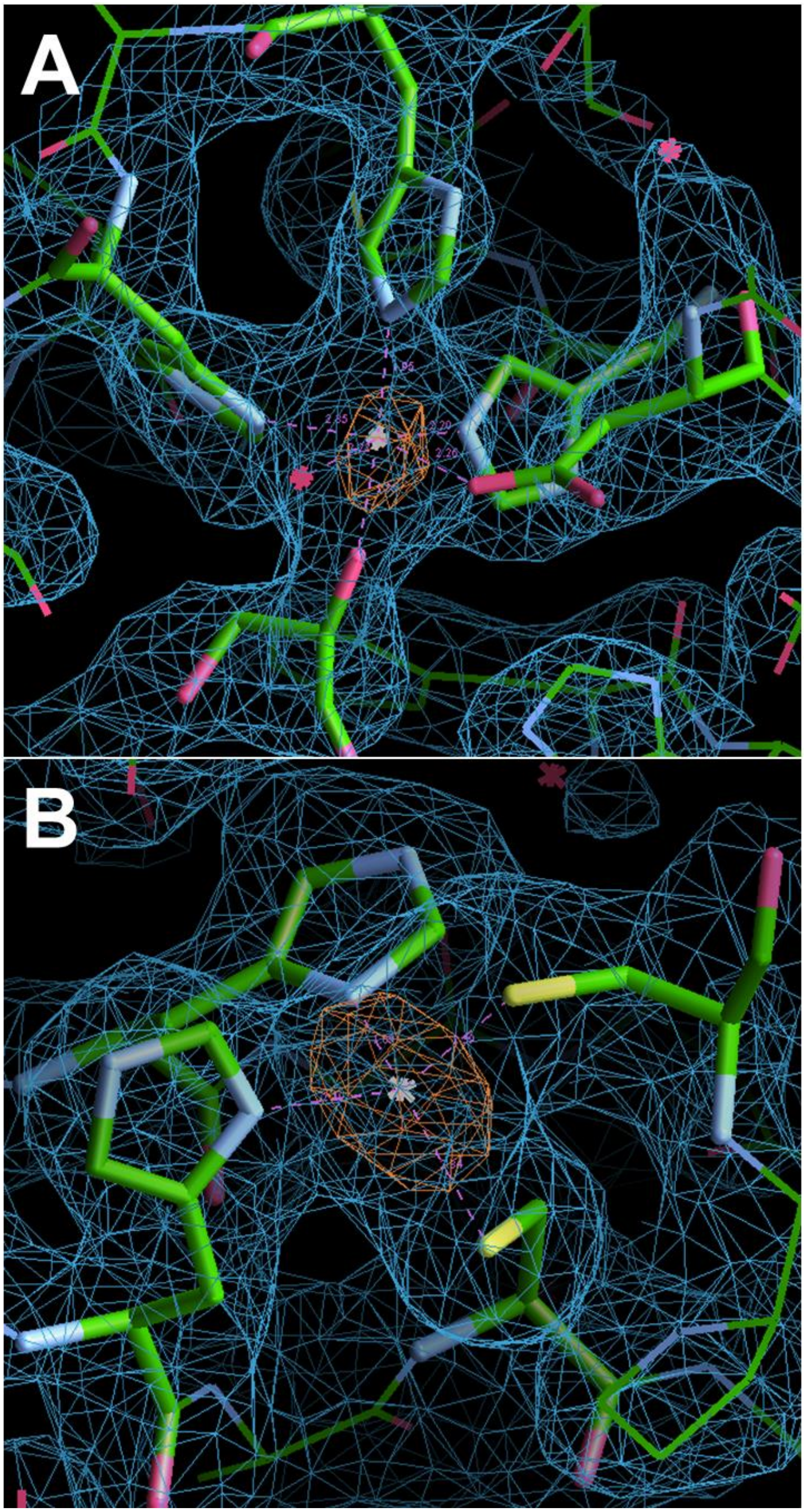
A
B

Figure S3 (A) Experimental map (blue) contoured at 1.0 σ and anomolous map (orange) contoured at 15.0 σ of the zinc ion in the active site of the Metallo-β-Lactamase domain. **(B)** Experimental map (blue) contoured at 1.0 σ and anomolous map (orange) contoured at 15.0 σ of the zinc ion in the novel, structural binding site in the β-CASP domain.
